# The Genetic and Phenotypic Diversity of *Bacillus* spp. from the Mariculture System in China and Their Potential Function against Pathogenic *Vibrio*

**DOI:** 10.3390/md21040228

**Published:** 2023-03-31

**Authors:** Yongxiang Yu, Yang Zhang, Yingeng Wang, Meijie Liao, Bin Li, Xiaojun Rong, Chunyuan Wang, Jianlong Ge, Jinjin Wang, Zheng Zhang

**Affiliations:** 1 Key Laboratory of Maricultural Organism Disease Control, Yellow Sea Fisheries Research Institute, Chinese Academic of Fishery Sciences, Qingdao 266071, China; yuyx@ysfri.ac.cn (Y.Y.);; 2 Laboratory for Marine Fisheries Science and Food Production Processes, Qingdao National Laboratory for Marine Science and Technology, Qingdao 266071, China; 3 College of Fisheries and Life Science, Shanghai Ocean University, Shanghai 201306, China

**Keywords:** *Bacillus*, antibiotics resistance, virulence, antibacterial ability, *Vibrio*, probiotic

## Abstract

*Bacillus* spp. could be one of the most suitable substitutes for the control and prevention of aquatic diseases. The occurrence of species population, antimicrobial character, and virulence diversity in *Bacillus* spp. recovered from the mariculture system in China between 2009 and 2021 were investigated, screening for probiotic *Bacillus* strains with good biological safety that can inhibit *Vibrio parahaemolyticus*, *V. alginolyticus*, *V. harveyi*, *V. owensii*, *V. campbellii*. The results showed that 116 *Bacillus* isolates were divided into 24 species, and the top three species were *B. subtilis* (37/116), *B. velezensis* (28/116), and *B. amyloliquefaciens* (10/116). Among the 116 *Bacillus* isolates, 32.8% were effective against *V. parahaemolyticus*, 30.1% for *V. alginolyticus*, 60.3% for *V. harveyi*, 69.8% for *V. owensii* and 74.1% for *V. campbellii*. More than 62% of *Bacillus* isolates were susceptible to florfenicol, doxycycline and tetracycline, etc., and 26/116 *Bacillus* isolates were found to be multiple-antibiotic-resistant (MAR), with MARI values ranging from 0 to 0.06. Eighteen kinds of antibiotic resistance genes were tested; only *tetB*, *blaTEM*, and *blaZ* were detected. And 9 isolates in 2 *Bacillus* species were excluded by 6/10 kinds of *Bacillus*-related toxin gene (*hblA*, *hblC*, *nheB*, *nheC*, *entFM*, *cykK*). Bio-safety testing indicated that three kinds of probiotics were good probiotic candidates to prevent Vibriosis. These results provide comprehensive genetic diversity, potential risks, and probiotic characteristics of *Bacillus* in the mariculture system in China, and provide basic support for green and healthy development of aquatic industry.

## 1. Introduction

Aquatic products play an important role as a component of healthy and indispensable supplements to nutritional diets. The aquaculture output reach to 80 million tons in recent years [1]. However, the occurrence of diseases is gradually becoming a major issue for the aquaculture industry [2]. For instance, approximately 60% of the reduced production in shrimp aquaculture can be attributed to diseases, 20% of which are caused by bacterial diseases [3]. *Vibrio* spp. is a Gram-negative bacteria commonly found in ocean and estuarine environments. Vibriosis is regarded as the bacterial disease caused by *Vibrio* spp. in shellfish and finfish aquaculture and is also a main cause of mortality in cultured shrimp throughout the world [4]. Pathogenic *Vibrio* spp. has been reported to cause foodborne diseases associated with seafood consumption. Meanwhile, Vibriosis in shrimps has been reported by many researchers, and at least 14 *Vibrio* species have been deemed responsible, including *V. parahaemolyticus*, *V. alginolyticus*, *V. harveyi*, *V. anguillarum*, *V. splendidus*, *V. mimicus*, *V. damsella*, *V*. *vulnificus*, *V. fischeri*, *V. cambellii*, *V. ordalli*, *V. mediterrani*, *V. orientalis* and *V. logei* [5,6,7].

The beneficial microbial strains referred to as probiotics are known to be “live microorganisms, when administered in adequate amounts, confer a health benefit on the host” [8]. Probiotics hold potential for use as effective drugs against aquaculture pathogens. *Bacillus* spp. is a commonly used probiotics in aquaculture. The application of *Bacillus* spp. could provide the most suitable substitute for the control and prevention of animal diseases. The majority of *Bacillus* species used as probiotics in aquaculture include *B. subtilis*, *B. licheniformis*, *B. pumilus*, *B. amyloliquefaciens,* etc. [6]. After *Bacillus* supplementation, increased resistance has been recorded against *V. parahaemolyticus*, *Edwardsiella tarda* [9], *Aeromonas salmonicida*, *Lactococcus garvieae*, *Streptococcus iniae* [10], *A*. *hydrophila* [11], *Acinetobacter* spp. [12]. Furthermore, improved disease resistance through the administration of dietary *Bacillus* has been reported in various aquatic species, such as rainbow trout [13], tilapia [14], and white shrimp [15].

On the other hand, certain *Bacillus* may cause aquatic diseases; for example, *B. cereus* is known as an important foodborne pathogen that can cause distinct forms of illness [16]. The occurrence of bacterial white spot syndrome (BWSS) may be associated with the unconventional use of probiotics containing *B. subtilis* in shrimp ponds [17]. Not only that, the probiotic bacterial species may present resistant strains and carry the risk of becoming conduits for the spread of toxin genes and antibiotic-resistance genes [18]. Therefore, only antibiotic-resistance and toxin-free candidate species should be considered for inclusion in probiotics [19].

At present, various genetic diversity studies have been conducted regarding the use of *Bacillus* in ecology, biotechnology, and industry [20,21]. However, poor-quality *Bacillus* is found in aquaculture, and systematic research on the ecological risk assessment of *Bacillus* is almost completely lacking. *Bacillus* leads to the release of pathogenic *Bacillus*, increasing environmental ecological risks and risks to the health of farmed animals. Based on this, *Bacillus* spp. isolates were collected from different aquatic environments in China between 2009 and 2021 for this study, and the genetic diversity, antibacterial and antibiotic properties, virulence, and antibiotics resistance genotype of the *Bacillus* spp. isolates were evaluated. Then, candidate strains with multi-pathogen antagonism and environmental friendliness were used for bio-safety testing. The results will provide a basic theoretical basis for the healthy use of *Bacillus* in mariculture production and to provide high-quality products for the aquaculture industry. This will promote the healthy, green, and sustainable development of the aquaculture industry.

## 2. Results

### 2.1. Bacillus Isolates Information

A total of 116 *Bacillus* isolates were activated and purified from the mariculture system. The collected *Bacillus* were identified on the basis of morphological, physiological, and biochemical characterizations as well as 16S rDNA and *re*c*A* sequencing. Sequencing analysis classified 116 *Bacillus* isolates into 24 different species (Table 1), including *B. subtilis* (37/116), *B. velezensis* (28/116), *B. amyloliquefaciens*(10/116), *B. stercoris* (6/116), *B. cereus* (6/116), *B. thuringiensis* (3/116), *B. megaterium* (3/116), *B. flexus* (3/116), *B. nealsonii* (2/116), *B. altitudinis* (2/116), *B. aryabhattai* (2/116), *B. atrophaeus* (2/116), *B. tequilensis* (1/116), *B. inaquosorum* (1/116), *B. stratosphericus* (1/116), *B. koreensis* (1/116), *B. lehensis* (1/116), *B. gibsonii* (1/116), *B. methylotrophicus* (1/116), *B. licheniformis* (1/116), *B. pumilus* (1/116), *B. haikouensis* (1/116), *B. circulans* (1/116), *B. marisflavi* (1/116).

Strains were photographed for colony morphology characteristics, and different morphotypes were discovered in 24 *Bacillus* species. Six *Bacillus* species showed that the colony was obtained in dried spore form with irregular edges, including *B. subtilis*, *B. velezensis*, *B. amyloliquefaciens, B. tequilensis*, *B. haikouensis*. Eighteen *Bacillus* species showed a moist and smooth morphology with regular edges, including *B.stercoris*, *B. cereus*, *B. thuringiensis*, *B. megaterium*, *B. flexus*, *B. nealsonii*, *B. altitudinis*, *B. aryabhattai*, *B. atrophaeus*, *B.inaquosorum*, *B. stratosphericus*, *B. koreensis*, *B. lehensis*, *B. gibsonii*, *B. methylotrophicus*, *B. pumilus*, *B. circulans*, *B. marisflavi*. Single-colony photographs of 24 *Bacillus* species are shown in Figure S1.

### 2.2. Antibacterial Activity Characterization of Bacillus Isolates against 5 Species of Vibrio

**Figure 1:**
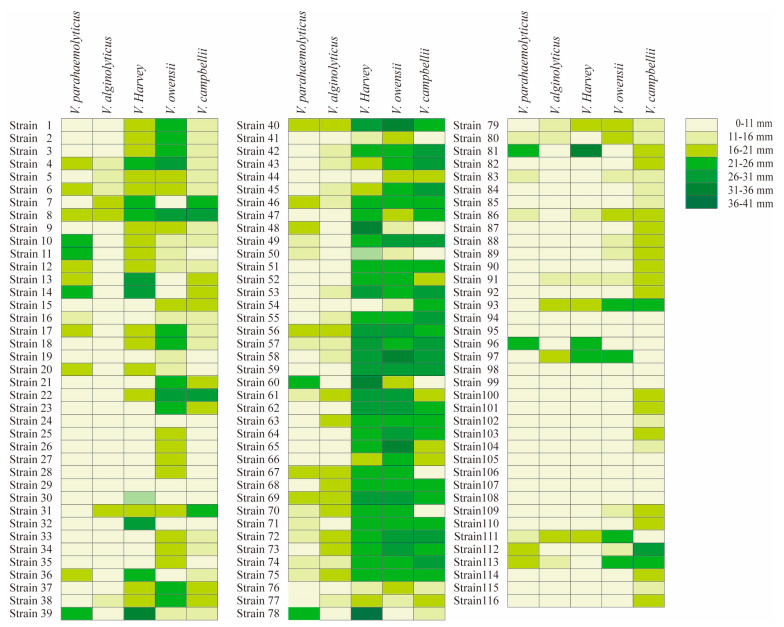
Inhibition zone of 116 *Bacillus* isolates under five kinds of *Vibrio*. The left is the strain number, and the right is the antibacterial diameter character. The colors from light to dark represent 0–11 mm, 11–16 mm, 16–21 mm, 21–26 mm, 26–31 mm, 31–36 mm and 36–41 mm repectively. Strains 1–37 were *B. subtilis*, strains 38–65 were *B. velezens*, strains 66–75 were *B.amyloliquefaciens*, strains 76–81 were *B. stercoris*, strains 82–87 were *B. cereus*, strains 88–90 were *B. thuringiensis*, strain 91 was *B. tequilensis*, strains 92–93 were *B. atrophaeus*, strains 94–95 were *B.nealsonii*, strain 96 was *B. inaquosorum*, strain 97 was *B. stratosphericus*, strains 98–100 were *B. flexus*, strains 101–103 were *B. megaterium*, strain 104 was *B. koreensis*, strains 105–106 were *B. aryabhattai*, strain 107 was *B. lehensis*, strain 108 was *B.gibsonii*, strain 109 was *B. methylotrophicus*, strain 110 was *B. licheniformis*, strain 111 was *B. pumilus*, strains 112–113 were *B. altitudinis*, strain 114 was *B. haikouensis*, strain 115 was *B. circulans*, strain 116 was *B. marisflavi*.

*Bacillus* isolates were tested on 5 species of *Vibrio*, and displayed typically different antibacterial ability among *Vibrio* species (Figure 1). The criterion of antibacterial activity was the presence of an inhibition zone, and the antibacterial ability showed typical differences among *Bacillus* isolates. Some probiotics had good antibacterial effects, and obvious inhibition zones can be observed. Among the 116 *Bacillus* isolates, 38 isolates were effective against *V. parahaemolyticus*, 35 isolates against *V. alginolyticus*, 70 isolates against *V. harveyi*, 81 isolates against *V*. *owensii* and 86 isolates against *V. campbellii* (Table 2).

Furthermore, in terms of *Vibrio* species, the antibacterial ability of *Bacillus* isolates showed differences among species and isolates. For the 38 isolates that were effective against *V. parahaemolyticus*, there were 12 *B. subtilis* isolates, 10 *B. velezensis* isolates, 7 *B. amyloliquefaciens* isolates, 3 *B. stercoris* isolates, 2 *B. altitudinis* isolates, 2 *B. cereus* isolates, 1 *B. pumilus* isolates, and 1 *B. inaquosorum* isolates. For the 35 isolates that were effective against *V. alginolyticus*, there were 6 *B. subtilis* isolates, 13 *B. velezensis* isolates, 8 *B. amyloliquefaciens* isolates, 3 *B. stercoris* isolates, 1 *B. tequilensis*, *B. atrophaeus*, *B. stratosphericus*, *B. pumilus*, *and B. altitudinis* isolates.

Among the 70 strains against *V. harveyi*, there were 23 *B. subtilis* isolates, 26 *B. velezensis* isolates, 10 *B. amyloliquefaciens* isolates, 1 *B. cereus*, *B. tequilensis*, *B. atrophaeus*, *B. stratosphericus*, *B. pumilus* and *B. inaquosorum* isolates. Among the 81 strains that against *V. owensii*, there were 37 *B. subtilis* isolates, 28 *B. velezensis* isolates, 10 *B. amyloliquefaciens* isolates, 3 *B. stercoris* isolates, 2 *B.cereus* and *B. altitudinis* isolates, 1 *B. tequilensis*, *B. atrophaeus*, *B. stratosphericus*, *B. pumilus*, *B. thuringiensis* and *B. methylotrophicus* isolates. Among the 81 strains that against *V. campbellii*, there were 37 *B. subtilis* isolates, 28 *B. velezensis* isolates, 8 *B. amyloliquefaciens* isolates, 6 *B. stercoris* isolates, 6 *B. cereus* isolates, 3 *B. megaterium* isolates, 2 *B. thuringiensis*, *B. altitudinis* and *B. atrophaeus* isolates, 1 *B. tequilensis*, *B. flexa*, *B. haikouensis*, *B. licheniformis* and *B. methylotrophicus*, *B. circulans*, *B. marisflavi* and *B. koreensis* isolates.

In addition, for the homogeneous *Bacillus* species, different effects were observed for the same type of *Vibrio* species. Among the 37 *B. subtilis* isolates, 25 isolates had no antibacterial effect on *V. parahaemolyticus*, and the zones of the remaining 12 isolates were 13.2–22 mm; 31 isolates had no antibacterial effect on *V. alginolyticus*, and the inhibition zone of the remaining 6 isolates was 14–20.9 mm; 14 isolates had no antibacterial effect on *V. harveyi*, and the inhibition zone of remaining 23 isolates was16.4–27.4 mm; 8 isolates had no antibacterial effect on *V*. *owensii*, and the inhibition zone of the remaining 29 isolates was 14.3–29.1 mm; 12 isolates had no antibacterial effect on *V. campbellii*, and the inhibition zone of the remaining 25 isolates was 14.3–29.6 mm.

For *B. velezensis*, among the 28 *B. velezensis*, 18 isolates had no effect on *V. parahaemolyticus*, and the inhibition zones of the remaining 10 isolates ranged from 13.8 to 21.7 mm; 15 strains had no effect on *V. alginolyticus*, and the diameter range of remaining 13 isolates ranged from 14.3 to 18.5 mm; for the *V. harveyi*, only 2 strains had no effective, remining isolates. The maximum diameter could reach 33.8 mm, while 14.7 is the minimum diameter. All 28 isolates were effective on *V*. *owensii*, from 11.7 to 33.4 mm; 4 isolates had no effect on *V. campbellii*, the diameter of the remaining 24 isolates ranged from 14.2 to 29.2 mm.

For *B. amyloliquefaciens*, among the 10 *B. amyloliquefaciens*, 3 strains had no effect on *V. parahaemolyticus*, and the inhibition zones of the remaining 7 isolates ranged from 13 to 19.7 mm; 2 strains had no effect on *V. alginolyticus*, and the inhibition zone of the remining 8 isolates was 14.7–19.7 mm; 10 strains had effective on *V. harveyi*, from 20.3–26.8 mm; 10 strains were effective on *V*. *owensii*, ranging from 22.2 to 27.9 mm; only 2 strains were effective for *V. campbellii*. The inhibition zone of the remining eight isolates was 20.3–27.1 mm.

### 2.3. Antibiotic Susceptibility Characterization of Different Kinds of Bacillus Isolates

**Figure 2:**
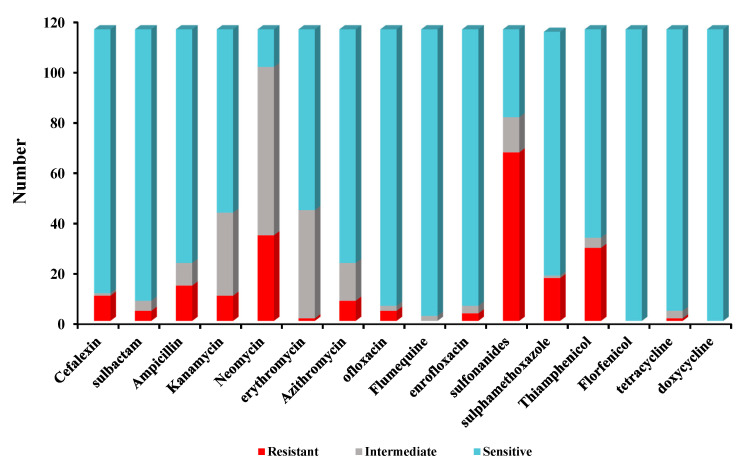
The antimicrobial susceptibility of 116 *Bacillus* isolates for 16 antibiotics.

Antimicrobial susceptibility tests indicated that high sulfadiazine resistance (67/116) was observed in 116 isolates. However, high susceptibility was observed to florfenicol (116/116), doxycycline (116/116), sulfamethoxazole-trimethoprim (114/116), tetracycline (112/116), ofloxacin (110/116), enrofloxacin (110/116), sulbactam (108/116), cefalexin (105/116), sulfamethoxazole (97/116), ampicillin (93/116), azithromycin (93/116), thiamphenicol (83/116). Low susceptibility to kanamycin (73/116), erythromycin (72/116), neomycin (15/116) was observed among 116 *Bacillus* isolates. The antimicrobial susceptibility for 116 *Bacillus* species against 16 antimicrobial agents are listed in Figure 2.

Furthermore, 26/116 *Bacillus* isolates were found to be multiple antibiotic resistant (MAR) and displaying resistance to at least three of the antibiotics tested in this study. The MARI of all the isolates ranged from 0 to 0.06. None of the tested isolates were resistant to all antibiotics at the same time. Two isolates (strain 7, strain 23) were resistant to seven types of antibiotics. The MAR of isolates resistant to 3 types of antibiotics was the most concentrated, and the proportion of isolates resistant to 4–6 types of drugs was lower (Table 3).

### 2.4. Distribution of Antimicrobial Resistance Genes among Bacillus Isolates

A total of 18 kinds of antimicrobial resistance genes were tested, but only 3 genes were detected (Figure S1), including the tetracycline gene *tetB*, which was detected in 116 isolates (100%), the chloramphenicol gene *cfr*, which was detected in 10/116 isolates (8.62%), and the β-lactams gene *blaTEM*, which was detected in 3/116 isolates (2.59%). The tetracycline resistance genes *tetA*, *tetC*, *tetD*; aminoglycoside resistance genes *ant*(*3′*)-*Ia*(*aadA*) and *aph*(*6′*)-*Id*(*strB*); quinolones genes *qnrA*, *qnrB*, *qnrS*, chloramphenicol genes *flor* and *cfr*; Sulfonamides genes *sul1*, *sul2*, *sul3*; and macrolides genes *ermA*, and *ermX* were not detected in any isolates.

Under 67 sulfonamides resistant isolates, none of the selected sulfonamides antimicrobial resistance genes were identified. All isolates were sensitive to tetracycline, but *tetB* was detected in 116 isolates. The *blaTEM* and *cfr* was detected some isolates that susceptibility to cefalexin, sulbactam, thiamphenicol and florfenicol.

### 2.5. Distribution of Virulence Genes among Bacillus Isolates

Based on the reported *Bacillus*-associated virulence genes, 10 kinds of virulence genes were detected in the *Bacillus* isolates. Only 9 of 116 isolates (7.8%) carried virulence-associated genes, of which 9/116 carried *entFM*, 7/116 carried *hblA* and *hblC*, 6/116 carried *nheB*, 5/116 carried *nheC* and *cytK*. The *hblD*, *nheA,* and *ces* genes were not detected in any strains. The positive, uniformly sized PCR products were sequenced and reconfirmed based on the NCBI database.

In addition, all virulence genes were detected in 2 *Bacillus* species (6 *B. cereus* isolates and 3 *B. thuringiensis* isolates); the *entFM* genes were found in 9 isolates (Figure S2); the detection rate was 100%; the *hbl*A and *hblC* were detected in 7 of 9 isolates; the *nheB* was found in 6/9 isolates; *nheC*, *cytK* and *bceT* were found in 5 of 9 isolates. The largest number of virulence-associated genes belonged to the *hblA*+*hblC*+*nheC*+*cytK*+*entFM*+*bceT* and *hblA*+*hblC*+*nheB+nheC*+*cytK*+*entFM* pattern.

### 2.6. Holistic Assessment of Potential Probiotics

Among the 116 *Bacillus* isolates, 12 *Bacillus* isolates were shown to have an inhibitory effect on 5 *Vibrio* species and can be used as potential probiotics to inhibit Vibriosis (Table 4), including 3 *B. subtilis* isolates, 5 *B. velezensis* isolates, and 4 *B. amyloliquefaciens* isolates. The largest inhibition zone for *V. parahaemolyticus* was 20.1 mm, and those for *V. alginolyticus, V. harveyi, V. owensii, V. campbellii* were 19.7 mm, 28.7 mm, 33.4 mm, and 28.7 mm, respectively.

**Figure 3:**
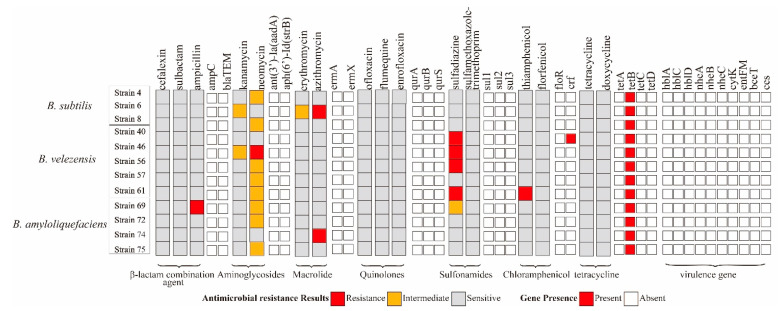
Gene presence and antibiotic susceptibility characteristics of 12 potential probiotics *Bacillus* isolates.

For antibiotic susceptibility characterization, *B. subtilis* strain 4, 6 was resistant to neomycin and azithromycin, and *B. velezens* strain 46, 61 was resistant to ampicillin and thiamphenicol. *B. velezens* strain 40, 46, 50, 61 was resistant to sulfonamides. Only *B. subtilis* strain 4 carried chloramphenicol gene *cfr*. The other 11 isolates contain 1 resistance gene *tetB* but were susceptible to tetracycline. All virulence genes were not detected in these strains (Figure 3).

### 2.7. Safety Testing of Potential Probiotics Bacillus Isolates in L. vannamei

**Figure 4:**
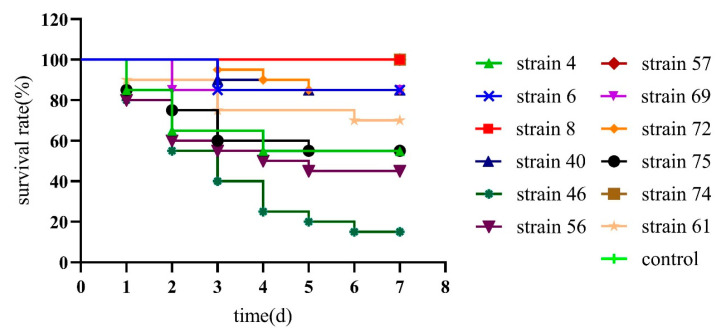
Survival curve of *Litopenaeus vannamei* after injected infection with 12 potential probiotics *Bacillus* isolates.

The 12 potential probiotics *Bacillus* suspension of 10^8^ CFU/mL was injected into the muscle of the second abdominal segment of *L. vannamei* and observed for 7 days to confirm the safety of the strains. The results showed that *B. subtilis* strain 4, 6, 40, *B. velezens* 46, 56, 61, and *B. amyloliquefaciens* 69, 72, 75 had pathogenic and lethal effects. *B. velezens* strain 46 mortality rates reached 85%, and the mortality rate of the remaining strains was between 15% and 55%. For the death experimental groups. Some minor mortality groups may be caused by the deterioration of the culture environment, rather than the pathogenicity of the strain. And the absolutely safety *Bacillus* isolates for *L. vannamei* were selected, 3 strains with obvious non-lethality were *B. subtilis* strain 8, *B. velezens* strain 57, and *B. amyloliquefaciens* strain 74 were a safe strain without death, and can be applied as a potential probiotic in *L. vannamei* (Figure 4).

## 3. Discussion

Biological antagonism aims to achieve the effect of “treating bacteria with bacteria” under natural conditions. Since *Pseudomonas bromoutilis* was isolated as antagonistic bacteria for the first time in 1966 [22], more and more antagonistic bacteria have been utilized. Probiotic bacteria isolated from the culture medium might exhibit antagonistic effects on present pathogens. *Bacillus* isolated from the marine environment can improve disease-resistant activity against bacterial infection [23]. It is a priority and inherently advantageous to use *B. pumilus* in the marine environment as a biocontrol agent or probiotic in aquaculture [24]. To form the basis of morphological characterizations and gene sequencing, the study reflected the diversity of *Bacillus* in our aquaculture systems. A total of 116 *Bacillus* isolates were categorized into 24 different species, including *B. subtilis*, *B. velezensis*, *B. amyloliquefaciens*, and *B. stercoris*, in this study. At present, Vibriosis has become an economically important disease in marine culture and adversely affected many cultured animals [25]. In the shrimp aquaculture system, *V. harveyi*, *V. alginolyticus* and *V. parahaemolyticus* are most frequently isolated [26]. *V. campbellii*, *V*. *owensii* were also pathogens of shrimp acute hepatopancreatic necrosis disease [27,28]. In this study, different *Bacillus* isolates had a variety of effects and also showed interspecific differences in pathogen inhibition effects. In addition, for the homogeneous *Bacillus* species, different effects were shown on the same type of *Vibrio* isolate. In this regard, the isolation of *Bacillus* from the mariculture system may have provided these isolates with an advantage in the inhibition of pathogens isolated from the marine environment.

According to the guidelines suggested by the European Food Safety Authority, this is essential for the absence of virulence factors in probiotic strains [29]. The presence of toxin genes in probiotic strains could potentially contribute to the prevalence of toxin genes among bacteria through horizontal transfer in some cases. To ensure the safe use of probiotic *Bacillus*, a safety evaluation must be carried out, common virulence genes must be screened, and the use of strains carrying virulence genes should be reduced as much as possible. In recent years, studies have been focused on the toxin genes character in probiotic *Bacillus* spp. Fu et al. [19] found that surveillance of anthrax toxin revealed that *cya* was detected in 8 of 31 farms. Almost half of the isolates could produce enterotoxins and various cytotoxic surfactin-like toxins in Cui et al. [30,31] report, and they summarized the state of current knowledge about the toxins of *B, cereus sensu lato* to be considered for safety assessment of probiotic candidates. Among the 116 *Bacillus* spp. isolates, very few isolates detected the presence of virulence genes, and 9 isolates of *Bacillus* can be excluded by virulence genes’ testing. Five isolates (4.3%) were tested as positive for hemolytic cytotoxin K (*cytK*), which is responsible for severe food poisoning. None of the species were positive for the emetic toxin gene *ces*, and the prevalence of *nheABC* and *hblCDA* was shown to be rare in the comparison [30,32]. Hemolytic activity assays are considered to be an important screening process. Hemolysin is a very common virulence factor, which frequently causes anemia and edema in the host; hence, hemolytic strains are not recommended for use as feed additives. The non-hemolytic strains would be preferable for probiotic use [33]. Generally, *nheABC* and *hblCDA* genes were widely distributed among *B. cereus* isolates from various origins, except for probiotic origin [30]. Deng et al. [34] suggested that *nheABC* and *hblCDA* were gradually prevalent in probiotic *Bacillus* spp. isolates. The current observations suggest that *hblC* and *nheA* were detected in *B. cereus* and *B. thuringiensis* isolates. No virulence genes were detected in 12 potential probiotics isolates.

Antibiotic resistance is one of the imminent challenges to global health with the current trend of escalating environmental contaminants; furthermore, the natural environment is a potential repository for the spread of antibiotic resistance [35]. Antibiotic susceptibility is one of the most important features of probiotic bacteria [36]. Reacher Yaylaci et al. [37] reported that *B. pumilus* was susceptible to furazolidone, erythromycin, ampicillin, oxytetracycline, rifampicin, and ciprofoxacin. Sorokulova et al. [38] previously reported that *Bacillus* spp. may be resistant to chloramphenicol. Our study is not similar in terms of results: 116 strains of *Bacillus* isolates showed low resistance to antibiotics, and more than 62% isolates were susceptible to florfenicol, doxycycline, sulfamethoxazole-trimethoprim, tetracycline, ofloxacin, enrofloxacin, sulbactam, cefalexin, sulfamethoxazole, ampicillin, azithromycin, thiamphenicol, kanamycin and erythromycin. The MARI reflects the degree of the environmental pollution caused by antibiotics that may be dangerous to human health [39]. A value of higher than 0.2 indicates a high antibiotic exposure risk, while a value lower than 0.2 indicates a low antibiotic exposure risk [40]. The MARI values of the 116 *Bacillus* isolates ranged from 0 to 0.06, which indicates a low antibiotic exposure risk.

Furthermore, the antibiotic resistance burden has serious implications for human health owing to the potential transfer of antibiotic resistance genes between bacteria, thereby impairing the efficacy of antibiotic treatment and compromising public health [41]. A total of 18 kinds of antibiotic resistance genes were tested, and only *tetB*, *blaTEM*, and *blaZ* were detected in this study. The previous studies indicated that the majority of *Bacillus* spp. for probiotics exhibited resistance to tetracycline, which mainly resulted in mobile tetracycline-resistance genes such as *tetB* and *tet45* [19,31,32]. In this study, 112 *Bacillus* isolates showed an antibiotics phenotype that was sensitive to tetracycline, but the *tetB* detection rate was 100%. Many researchers have observed discrepancies between antimicrobial resistance phenotypes and the prevalence of antimicrobial resistance genes [42,43]. This might be due to the antimicrobial phenotypes that can be expressed upon the stimulation of many different genetic determinants [44]. The macrolide-resistance genes present on extrachromosomal elements have been identified in mobile elements, such as the plasmid-encoded *ermC* from *B. subtilis* [45]. Tetracycline-resistance determinants have also been found in mobile elements, including the plasmid-encoded *tetL* gene from *B. subtilis* [46], and the *tetM* contained within the conjugative transposon Tn5397 of *B. subtilis* [47]. Other tetracycline resistance genes, such as *tetK*, have been observed in some *Bacillus* isolates [48]. The detection of chloramphenicol gene cfr (8.62%) and β-lactams gene *bla*TEM (2.59%) was low. Chloramphenicol resistance can be attributed to the enzymatic inhibition of the drug facilitated by chloramphenicol acetyl-transferases [49]. Beta lactams are the most broadly used class of antimicrobial agents, characterized by minimal toxicity and employed in the treatment of various bacterial ailments, including those attributed with different *Vibrio* species [50]. Apparently, resistance among the beta-lactam and chloramphenicol drugs was low.

## 4. Materials and Methods

### 4.1. Bacterial Strains and Growth Condition

A total of 116 *Bacillus* isolates were collected from a mariculture system in China with geographic and chronological differences between 2009 and 2021. All samples were cultured on tryptone soybean broth (TSB) agar medium at 28 °C for 36 h. Single colonies were picked and inoculated on TSB medium to obtain purified bacteria. Then single colony was picked up with a tip under sterile conditions, transferred to 100 μL ultrapure water and repeatedly pipetted and, after 12 min in a metal bath at 99 °C, centrifuged for 5 min at 4 °C and 12,000 rpm with a high-speed centrifuge, the supernatant was taken. The DNA concentration was determined in the supernatant using a spectrophotometer (Nano Drop 1000) as template DNA in PCR. The PCR system consists of 1 μL of DNA template, 25 μL of Taq Master Mix (Vazyme, Nanjing, China), 1 μL of forward primers and 1 μL of reverse primers, and double-distilled water for a total volume of 50 μL. PCR amplification conditions were: 94 °C, 4 min; 30 cycles: 94 °C, 30 s, 55 °C, 30 s, 72 °C, 1.5 min; store at 72 °C, 10 min, 4 °C. PCR products were sequenced after detection by 1% agarose gel electrophoresis. Primer synthesis and gene sequencing were completed by Sangon Biotech (Shanghai, China). Colonies were preliminarily identified by morphology and 16S rDNA sequencing. BLAST was used for nucleotide comparison to obtain percentage similarity. The sequence analysis was carried out based on *recA* sequence to more accurately identify the dominant bacteria, as described by Mohkam.

Five common pathogenic *Vibrio* spp. of *Litopenaeus vannamei*, including *V. parahaemolyticus*, *V. alginolyticus*, *V. harvey*, *V. owensii*, and *V. campbellii*, were tested to observe the antibacterial effect of *Bacillus* isolates. These strains were activated and cultured at 28 °C for 24 h in TSB for activation.

### 4.2. Antibacterial Ability of Bacillus Isolates against Vibrio spp.

Antibacterial activity was assessed using the agar well diffusion assay method. The *Bacillus* and *Vibrio* isolates were cultured in TSB media at 28 °C, 180 rpm for 24 h. Then, the bacteria suspension was adjusted to 1 × 10^9^ CFU/mL for *Bacillus*, 1 × 10^6^ CFU/mL for *Vibrio*. 100 μL *Vibrio* suspension was smeared on the surface of TSB medium and 100 μL of *Bacillus* suspension was added into the hole and cultured at 28 °C. After 36 h, checks were carried out for the appearance of inhibition haloes surrounding the putative antagonists’ spots and the diameter of the inhibition zone was measured with Scan 1200 inhibition zone reader function (Interscience, Saint-Nom-la-Bretèche, France) to screen the beneficial strains with good antibacterial effect. All the experiments were performed in triplicate.

### 4.3. Antibiotic Susceptibility Test

The antimicrobial resistance of 116 *Bacillus* isolates was determined using the Kirby–Bauer disk diffusion method on TSB agar medium according to the guidelines by the National Committee for Clinical Laboratory Standards with minor changes [51]. A total of 16 kinds of antibiotics were used in this study, including thiamphenicol, erythromycin, kanamycin, ofloxacin, florfenicol, azithromycin, cefalexin, flumequine, sulfamethoxazole–trimethoprim, enrofloxacin, tetracycline, sulfadiazine, neomycin, sulbactam, ampicillin, and doxycycline. *Escherichia coli* ATCC 25922 was used for quality control. The plates were incubated at 28 °C for 24 h. All the experiments were performed in triplicate. Then, the antibiotic susceptibility was reported as resistant (R), intermediate (I) and sensitive (S) based on the inhibitory zone diameter according to the CLSI standards.

Furthermore, the isolates were considered multiple-antibiotic resistant (MAR) if the isolate was resistant to three or more separate antimicrobial classes [52]. The MAR and multiple antibiotic resistance index (MARI) were measured for all isolates according to the CLSI protocol [53].

### 4.4. Antimicrobial Resistance Genes Detection among Bacillus Isolates

18 antimicrobial resistance genes belong to 7 categories were studied using PCR amplification, including tetracycline resistance (*tetA*, *tetB*, *tetE*), extended-spectrum β-lactamase (ESBL) (*blaTEM*, *ampC*, *blaZ*), aminoglycoside resistance [*ant*(*3*)-*Ia*(*aadA*), *aph*(*6′*)-*Ib*(*strB*)], sulphonamide resistance (*sul1*, *sul2* and *sul3*), macrolides (*ermA*, *ermX*), and phenicols (*floR*, *cfr*), as well as quinolones (*qnrA*, *qnrB*, *qnrS*) resistance genes. Table 1 describes the primers and protocols used. Each assay was composed of 1 μL of DNA material, 12.5 μL of Taq Master Mix (Vazyme, China), 1 μL of forward and 1 μL of reverse primers, and double-distilled water to obtain a total volume of 25 μL. The PCR product was analyzed by electrophoresis using 1% (*w/v*) agarose gel and then visualized under UV transilluminator. The positive, uniformly sized PCR products were sequenced and reconfirmed based on the NCBI database.

### 4.5. Molecular Detection of Potential Virulence Gene among Bacillus Isolates

Based on Fu et al. [54], the presence of 10 kinds of *Bacillus*-related toxin genes was detected in 116 *Bacillus* isolates, including *hblA*, *hblC*, *hblD*, *nheA*, *nheB*, *nheC*, *cytK*, *entFM*, *bceT* and *ces*. The primers and the expected size of the DNA product for each of the investigated genes are shown in Table 5.

The primers were synthesized by Sangon Biotech (Qingdao, China). The DNA of the purified bacteria was extracted by the Beijing Tiangen biological bacterial genome DNA extraction kit. Extracted DNA material served as a template for PCR amplification for each tested sample. Each assay was composed of 1 μL of DNA material, 12.5 μL of Taq Master Mix (Vazyme, China), 1 μL of forward and 1 μL of reverse primers, and double-distilled water to obtain a total of 25 μL. The PCR product was analyzed by electrophoresis using a 1% (*w/v*) agarose gel and then visualized under a UV transilluminator. The positive bands’ sequence information was determined and compared to verify the accuracy of the sequence information.

### 4.6. Bio-Safety Testing in Litopenaeus vannamei

To evaluate the in vivo security of 12 potential probiotics *Bacillus* isolates, *L. vannamei* was used in this study. *Bacillus* were cultured in TSB and incubated with shaking for 36 h at 28 °C. Shrimp (5–8 cm) were randomly divided into 13 groups (12 groups for *Bacillus* infection and 1 group for control), with each group containing 20 shrimp. The shrimp were injected with 1.0 × 10^8^ CFU/mL *Bacillus* suspension, and PBS was injected as control. Then, mortality was monitored and recorded for 7 days.

### 4.7. Statistical Analysis

Data were preliminary analyzed by excel 2016 software, and all statistical analyses were carried out using SPSS software (version 26.0). The results are expressed as mean ± SE of mean (SEM) with One-way ANOVA at 5%. The colony photographs were adjusted with Adobe Illustrator 2019 to best represent the colony morphology. Survival curves were made by GraphPad.

## 5. Conclusions

Our study provides comprehensive diversity and shows the potential risks of the *Bacillus* species collected from geographically distinct regions in the mariculture system in China for the first time. The phenotype and genetic diversity of different kinds of *Bacillus* spp. were investigated. An integral evaluation of the virulence and antimicrobial resistance risk of *Bacillus* isolates was carried out. Then, the potential probiotics were screened with multiple antibacterial activities for different *Vibrio* species and evaluated the probiotic character. Finally, three potential probiotic strains were found. These results provide basic support for microecological preparations in aquatic disease preservation and promote the healthy and green development of aquatic industry.

## Figures and Tables

**Table 1:** Type and quantity of *Bacillus* isolates.

Isolates	Numbers	Isolates	Numbers
*B. subtilis*	37	*B. tequilensis*	1
*B. velezensis*	28	*B.inaquosorum*	1
*B. amyloliquefaciens*	10	*B. stratosphericus*	1
*B.stercoris*	6	*B. koreensis*	1
*B. cereus*	6	*B. lehensis*	1
*B. thuringiensis*	3	*B. gibsonii*	1
*B. megaterium*	3	*B. methylotrophicus*	1
*B. flexus*	3	*B. licheniformis*	1
*B. nealsonii*	2	*B. pumilus*	1
*B. altitudinis*	2	*B. haikouensis*	1
*B. aryabhattai*	2	*B. circulans*	1
*B. atrophaeus*	2	*B. marisflavi*	1

**Table 2:** Cluster of the *Bacillus* isolates that inhibit 5 pathogenic bacteria.

Pathogenic Bacterial	*Bacillus* Isolates Numbers
*V. parahaemolyticus*	38
*V. alginolyticus*	35
*V. harvey*	70
*V. owensii*	81
*V. campbellii*	86

**Table 3:** Multiple antibiotic resistance (MAR) index of *Bacillus* isolates.

Multiple Antibiotic Resistance	% of Occurrence
MAR (3)	8.62
MAR (4)	4.31
MAR (5)	6.03
MAR (6)	1.72
MAR (7)	1.72

Note: MAR (3) represent strains resisting 3 or more antibiotics.

**Table 4:** Antibacterial character of 12 potential probiotics *Bacillus* isolates against 5 *Vibrio*.

Strains No.	Inhibition Zone (mm) *				
	*V. parahaemolyticus*	*V. alginolyticus*	*V. harvey*	*V. owensii*	*V. campbellii*
strain 4	16.0 ± 0.73	15.7 ± 0.41	21.3 ± 0.49	28.3 ± 0.98	14.5 ± 0.49
strain 6	17.0 ± 0.90	14.0 ± 0.73	18.1 ± 0.57	19.3 ± 0.49	13.4 ± 0.65
strain 8	18.5 ± 0.41	18.4 ± 0.49	22.6 ± 0.16	27.8 ± 0.65	28.7 ± 0.89
strain 40	20.1 ± 0.73	18.5 ± 0.57	27.6 ± 0.24	33.4 ± 0.57	25.0 ± 0.57
strain 46	16.2 ± 0.24	14.9 ± 0.65	25.1 ± 0.49	24.7 ± 0.49	23.6 ± 0.41
strain 56	16.2 ± 0.98	17.4 ± 1.06	28.7 ± 0.33	30.9 ± 1.06	25.5 ± 0.65
strain 57	15.3 ± 0.57	15.3 ± 0.98	26.9 ± 0.82	24.7 ± 1.14	27.7 ± 0.98
strain 61	15.7 ± 0.65	16.4 ± 0.57	26.2 ± 0.98	30.7 ± 0.73	20.8 ± 0.90
strain 69	19.2 ± 0.90	19.7 ± 0.65	26.8 ± 0.41	27.9 ± 0.82	23.1 ± 0.73
strain 72	14.2 ± 0.73	17.7 ± 0.33	24.2 ± 0.65	27.5 ± 0.73	27.1 ± 1.22
strain 74	14.3 ± 0.57	14.7 ± 0.98	22.7 ± 0.98	25.2 ± 0.98	26.0 ± 0.57
strain 75	13.0 ± 0.98	16.6 ± 0.82	22.5 ± 0.49	24.3 ± 0.33	25.5 ± 0.73

* Values are represented as mean ± SEM (*n* = 3).

**Table 5:** PCR primers used to this study.

Primer	Sequence (5′-3′)	Gene	Amplicon Size (bp)	Reference
*hblA*-F	AAGCAATGGAATACAATGGG	*hblA*	1154	[54]
*hblA*-R	AGAATCTAAATCATGCCACTGC			
*hblC*-F	GATACTCAATGTGGGAACTGC	*hblC*	740	
*hblC*-R	TTGAGACTGCTCGTCTAGTTG			
*hblD*-F	ACCGGTAACACTATTCATGC	*hblD*	829	
*hblD*-R	GAGTCCATATGCTTAGATGC			
*nheA*-F	GTTAGGATCACAATCACCGC	*nheA*	755	
*nheA*-R	ACGAATGTAATTTGAGTCGC			
*nheB*-F	TTTAGTGGATCTGTACGC	*nheB*	743	
*nheB*-R	TTAATGTTCGTTAATCCTGC			
*nheC*-F	TGGATTCCAAGATGTAACG	*nheC*	683	
*nheC*-R	ATTACGACTTCTGCTTGTGC			
*cytK*-F	CGACGTCACAAGTTGTAACA	*cytK*	565	[55]
*cytK*-R	CGTGTGTAAATACCCAGTT			
*entFM*-F	GTTCGTTCAGGTGCTGGTAC	*entFM*	486	
*entFM*-R	AGCTGGGCCTGTACGTACTT			
*bceT*-F	TTACATTACCAGGACGTGCTT	*bceT*	428	[56]
*bceT*-R	TGTTTGTGATTGTAATTCAGG			
*ces*-F	GGTGACACATTATCATATAAGGTG	*ces*	1271	
*ces*-R	GTAAGCGAACCTGTCTGTAACAACA			
*tetA*-F	GCTACATCCTGCTTGCCTTC	*tetA*	212	[57]
*tetA*-R	GCATAGATCGCCGTGAAGAG			
*tetB*-F	TACGTGAATTTATTGCTTCGG	*tetB*	206	
*tetB*-R	ATACAGCATCCAAAGCGCAC			
*tetD*-F	TGTGCTGTGGATGTTGTATCTC	*tetD*	844	
*tetD*-R	CAGTGCCGTGCCAATCAG			
*qnrA*-F	TTCAGCAAGAGGATTTCTCA	*qnrA*	500	[58]
*qnrA*-R	GGCAGCACTATTACTCCCAA			
*qnrB*-F	CCTGAGCGGCACTGAATTTAT	*qnrB*	617	
*qnrB*-R	GTTTGCTGCTCGCCAGTCGA			
*qnrS*-F	ACATAAAGACTTAAGTGATC	*qnrS*	619	
*qnrS*-R	CAATTAGTCAGGATAAAC			
*floR*-F	CTGCTGATGGCTCCTTTC	*flor*	650	[59]
*floR*-R	GCCGTGGCGTAACAGAT			
*cfr*-F	TGAAGTATAAAGCAGGTTGGGAGTCA	*cfr*	746	
*cfr*-R	ACCATATAATTGACCACAAGCAGC			
*Sul1*-F	GTGACGGTGTTCGGCATTCT	*sul1*	800	[60]
*Sul1*-R	TCCGAGAAGGTGATTGCGCT			
*Sul2*-F	CATCATTTTCGGCATCGTC	*sul2*	793	
*Sul2*-R	TCTTGCGGTTTCTTTCAGC			
*Sul3*-F	GCAACAGTTGGTGCTAAACGAGA	*sul3*	578	
*Sul3*-R	AGCAGATGTGATTGATTTGGGAG			
*ant*(*3′*)-*Ia*(*aadA*)-F	ATCTGGCTATCTTGCTGACA	*ant*(*3′*)-*Ia*(*aadA*)	388	[61]
*ant*(*3′*)-*Ia*(*aadA*)-R	TTGGTGATCTCGCCTTTC			
*aph*(*6′*)-*Id*(*strB*)-F	ATGTTCATGCCGCCTGTTTTT	*aph*(*6′*)-*Id*(*strB*)	837	
*aph*(*6′*)*-Id*(*strB*)-R	CTAGTATGACGTCTGTCGC			
*ermA*-F	GTTCAAGAACAATCAATACAGAG	*ermA*	421	[62]
*ermA*-R	GGATCAGGAAAAGGACATTTTAC			
*ermX*-F	GTTGCGCTCTAACCGCTAAGGC	*ermX*	566	
*ermX*-R	CCATGGGGACCACTGAGCCGTC			
*blaTEM*	AAAGATGCTGAAGATCA	*blaTEM*	425	[63]
*blaTEM*	TTTGGTATGGCTTCATTC			
*ampC*	GCGAAAGCCAGCTGTCGGGC	*ampC*	550	[64]
*ampC*	CCYTTTTATGTACCCAYGA			
*blaZ*	ACTTCAACACCTGCTTTC	*blaZ*	490	
*blaZ*	TGACCACTTTTATCAGCAACC			
27F	AGAGTTTGATCCTGGCTCAG	16S rDNA	1542	[65]
1492R	TACGGCTACCTTGTTACGACTT			
*recA*-F	GATCGTCAAGCAGCCTTAGAT	*recA*	540	
*recA*-R	TTACCGACCATAACGCCGAC			

## Data Availability

The data that support the findings of this study are available from the corresponding author upon reasonable request.

## Supplementary Materials

The following supporting information can be downloaded at: https://www.mdpi.com/article/10.3390/md21040228/s1, Figure S1: Morphological diversity of representative *Bacillus* species. Figure S2: Antimicrobial resistance genes gel electrophoresis.; Figure S3: Virulence genes gel electrophoresis.
