# Supplementary material for: The Genetic and Phenotypic Diversity of Bacillus spp. from the Mariculture System in China and Their Potential Function against Pathogenic Vibrio
Source: Mar Drugs. 2023 Mar 31;21(4):228. doi: 10.3390/md21040228 (PMC10146669; doi:10.3390/md21040228)
Supplement: Supplementary file 1 [file marinedrugs-21-00228-s001.zip › marinedrugs-2258717-supplementary.pdf]

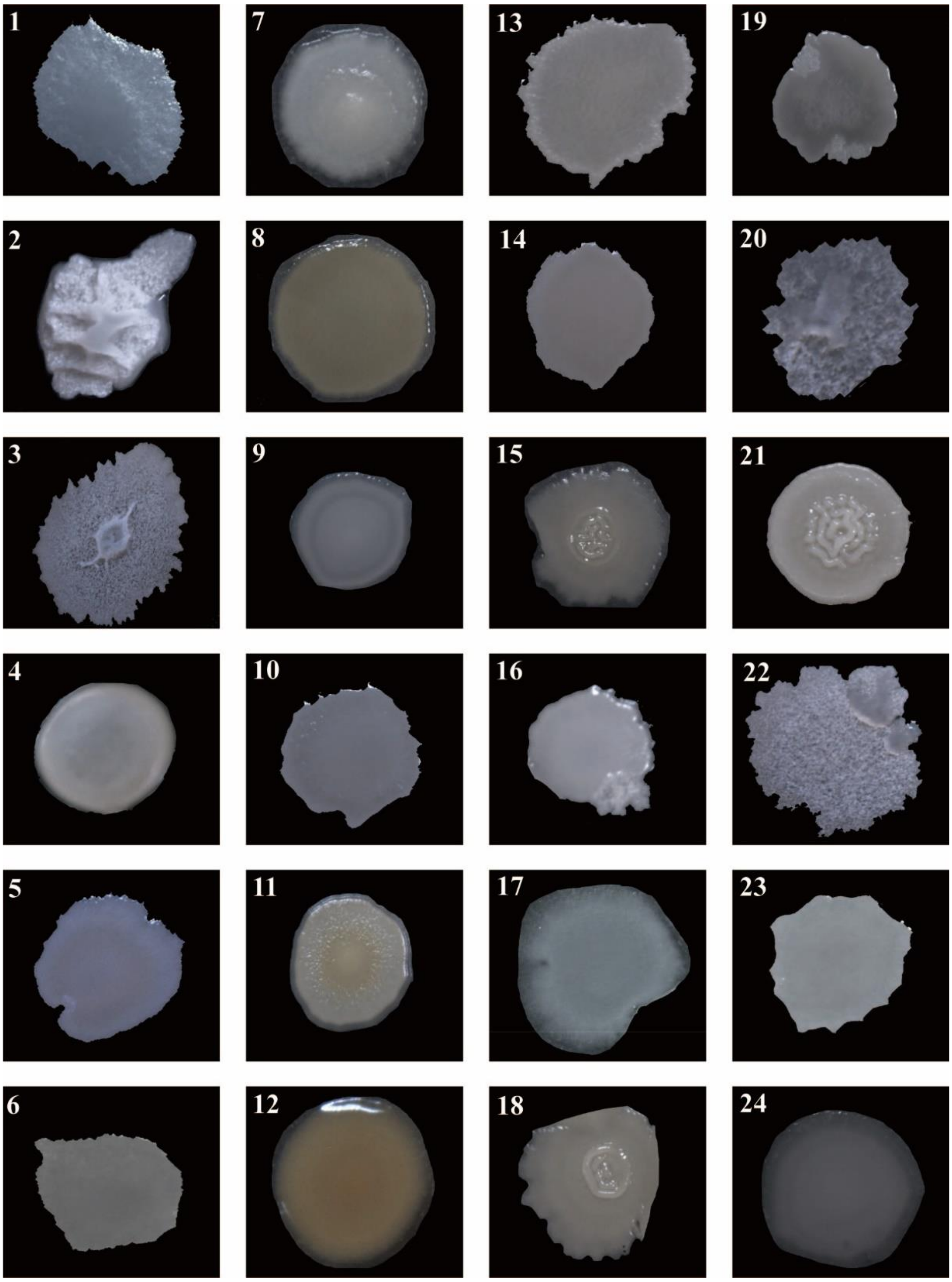

Figure S1. Morphological diversity of representative *Bacillus* species. Photographs of colonies grown for 36 h at 28℃ in TSB agar medium. Images were all at the same scale. The number represented in the top left corner represents the number of the morphotype on the photo, and No. 1-24 respectively present *B. subtilis*, *B. velezensis*, *B. amyloliquefaciens*, *B. stercoris*, *B. cereus*, *B. t*huringiensis, *B. megaterium*, *B. flexus*, *B. nealsonii*, *B. altitudinis*, *B. aryabhattai*, *B. atrophaeus*, *B. tequilensis*, *B. inaquosorum*, *B. stratosphericus*, *B. koreensis*, *B. lehensis*, *B. gibsonii*, *B. methylotrophicus*, *B. licheniformis*, *B. pumilus*, *B. haikouensis*, *B. circulans*, and *B. marisflavi*. Colonies are not in the same scale as some would appear smaller than others and would affect the visual separation into morphotypes. The colony size was between 1 mm to 5 mm in diameter.

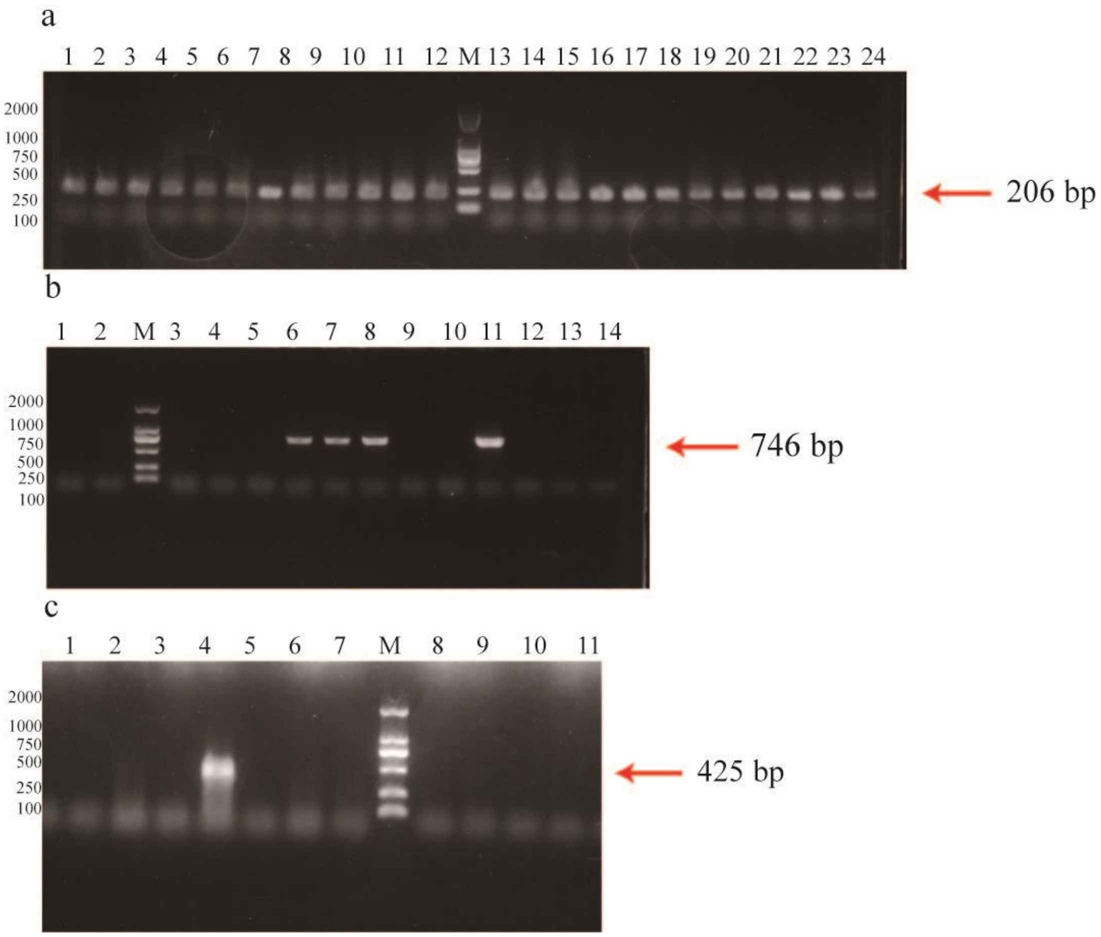

Figure S2. Antimicrobial resistance genes gel electrophoresis. (a) Gel electrophoresis showing the *tetB* among isolates belonging to *Bacillus*. Lane M: Molecular Marker (2000 bp); Lane 1-24: positive isolates of *tetB* (206 bp). (b) Gel electrophoresis showing the *cfr* among isolates belonging to *Bacillus*. Lane M: Molecular Marker (2000 bp), lane 1: Negative control; Lane 6-8, 11: positive isolates of *cfr* (746 bp); (c) Gel electrophoresis showing the *blaTEM* among isolates belonging to *Bacillus*. Lane M: Molecular Marker (2000 bp), lane 1: Negative control; Lane 4: positive isolates of *blaTEM* (425 bp).

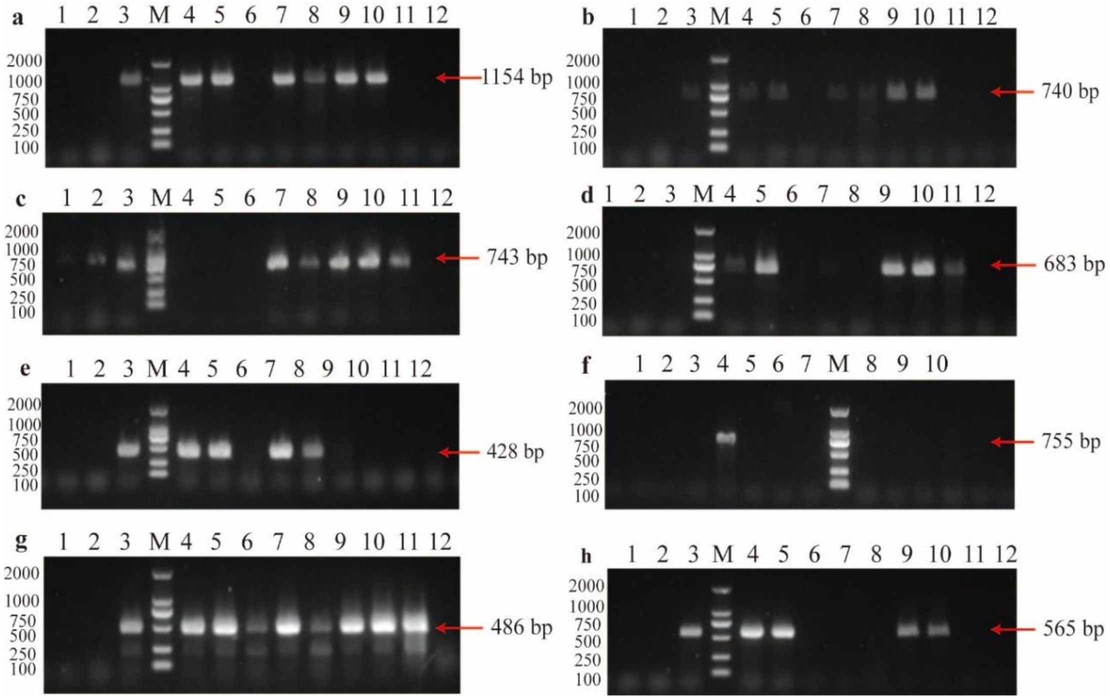

Figure S3. Virulence gene gel electrophoresis. (a) Gel electrophoresis showing the *hblA* among isolates belonging to *Bacillus*. Lane M: Molecular Marker (2000 bp); Lane 3-5, 7-10: positive isolates of *hblA* (1154 bp); (b) Gel electrophoresis showing the *hblC* among isolates belonging to *Bacillus*. Lane M: Molecular Marker (2000 bp); Lane 3-5, 7-10: positive isolates of *hblC* (740 bp); (c) Gel electrophoresis showing the *nheB* among isolates belonging to *Bacillus*. Lane M: Molecular Marker (2000 bp); Lane 2-3, 7-11: positive isolates of *nheB* (743 bp); (d) Gel electrophoresis showing the *nheC* among isolates belonging to *Bacillus*. Lane M: Molecular Marker (2000 bp); Lane 4-5, 9-11: positive isolates of *nheC* (683 bp); (e) Gel electrophoresis showing the *bceT* among isolates belonging to *Bacillus*. Lane M: Molecular Marker (2000 bp); Lane 3-5, 7-8: positive isolates of *bceT* (428 bp); (f) Gel electrophoresis showing the *nheA* among isolates belonging to *Bacillus*. Lane M: Molecular Marker (2000 bp); Lane 4: positive isolates of *nheA* (755 bp); (g) Gel electrophoresis showing the *entFM* among isolates belonging to *Bacillus*. Lane M: Molecular Marker (2000 bp); Lane 3-11: positive isolates of *entFM* (486 bp); (hH) Gel electrophoresis showing the *cytK* among isolates belonging to *Bacillus*. Lane M: Molecular Marker (2000 bp); Lane 3-5, 9-10: positive isolates of *cytK* (565 bp).
